# A 6-months assessment of the alcohol-related clinical burden at emergency rooms (ERs) in 11 acute care hospitals of an urban area in Germany

**DOI:** 10.1186/1472-6963-5-73

**Published:** 2005-11-18

**Authors:** Bernhard T Baune, Rafael T Mikolajczyk, Gerhard Reymann, Annette Duesterhaus, Susanne Fleck, Hildegard Kratz, Ulrike Sundermann

**Affiliations:** 1 Mental Health Epidemiology, Department of Psychiatry, University of Muenster, Germany; 2 School of Public Health, University of Bielefeld, Germany; 3 Clinic for Addiction, Westphalian Hospital Dortmund, University of Bochum, Germany; 4 City Council of Public Health, City of Dortmund, Germany

## Abstract

**Background:**

The purpose of the study was to identify and to profile alcohol-related attendances to emergency rooms (ERs) of 11 hospitals of various medical specialties covering a large urban population, to assess risk factors associated with short-stay cases, repeat attendances and higher degree of alcohol consumption and to estimate their impact on the alcohol-related burden at ERs.

**Methods:**

A 6-months study was carried out to obtain clinical and administrative data on single and multiple attendances at ERs in 11 governmental acute hospitals in a large city in Germany. All alcohol-related attendances at ERs of study hospitals were eligible. A broad definition of alcohol-related attendances independently from alcohol diagnosis and various demographic, clinical and administrative measures were used. Odds ratios for the associations of these measures with duration of stay, repeat attendances and higher degrees of alcohol consumption were derived from multivariate binomial and multinomial logistic regression models.

**Results:**

1,748 patients with symptoms of alcohol consumption or withdrawal (inclusion rate 83.8%) yielded 2,372 attendances (3% of all medical admissions), and resulted in 12,629 inpatient-days. These patients accounted for 10.7 cases per 1,000 inhabitants. The average duration of inpatient stay was 10 days. 1,451 of all patients (83%) presented once, whereas the median of repeat attendances was three for the remaining 297 patients. Short-stay cases (<24 hours) were significantly linked with male gender, alcohol misuse, trauma (or suspicion of a trauma) and medical specialties. Increased levels of alcohol consumption at first attendance were significantly associated with repeat attendances in due course. In a multinomial logistic regression model higher degrees of alcohol consumption were significantly associated with male gender, trauma, short-stays, attendance outside regular working time, and with repeat attendances and self-discharge.

**Conclusion:**

Apart from demographic factors, the alcohol-related clinical burden is largely determined by short-stay cases, repeat attendances and cases with higher levels of alcohol consumption at first attendance varying across medical specialties. These findings could be relevant for the planning of anti-alcoholic interventions at ERs.

## Background

Alcohol misuse constitutes a major problem in modern society and both physical and mental alcohol-related harm result in a large number of attendances at emergency rooms (ERs), imposing a significant burden on the workload and financial resources of the medical departments [1,2]. Excess alcohol consumption with attendance at ERs may occur as part of an alcohol or drug dependence, as a cause of trauma or as a co-morbid condition of psychiatric diseases [3-5]. Furthermore, alcohol-related withdrawal syndromes may play an important role in the management of patients attending ERs [6].

Most previous studies on the alcohol-related clinical burden of emergencies on health care settings were based on samples drawn from single practices, single hospitals or only certain departments (e.g. surgical, internal medicine, orthopaedics) of several hospitals [7-10]. Selection bias and lack of representativeness are often major limitations of these studies. Previous studies in this field also have reported on the impact of short stay cases and repeat cases on the clinical burden of ERs at general hospitals [8,11-13] and noted an increasing number of younger patients attending ERs in recent years in the UK and Ireland [14,15].

O'Farrell et al. reported in their study of an increase of alcohol-related admissions to ERs for each age group over time indicating a rising burden for hospitals between 1997–2001 in Ireland [15]. The importance of the alcohol-related clinical burden caused by younger people also has been demonstrated in a study performed in an inner-city hospital in the UK [14]. Hazardous alcohol consumption in the general population in Germany has increased in younger people aged 18–24 between the years 1995–2001 and has slightly decreased in older men and women in the general population in Germany [16]. It is unknown whether the increasing number of younger people with hazardous alcohol consumption in Germany receive alcohol specific interventions at all, and particularly present at ERs due to alcohol-related problems.

However, since duration of stay, number of attendances and degree of alcohol consumption are contributing to the alcohol-related burden at ERs, little knowledge has been obtained in previous studies whether this is true for a wide range of medical specialties in hospitals and which demographic and clinical factors are associated with these contributing factors. In this study, which should serve as a needs assessment of future alcohol-related interventions at ERs, a complete sample of alcohol-related attendances at ERs of all existing hospitals covering a wide range of medical specialties in an urban area of a known population size was assessed for the duration of 6 months.

### Objectives

The aims of the study were as follows:

1. To identify alcohol-related attendances at emergency rooms (ERs) of 11 hospitals of various medical specialties covering a large urban population;

2. To profile the alcohol-related attendances;

3. To assess sociodemographic and clinical risk factors associated with short-stay cases, repeat attendances and higher degree of alcohol consumption and to assess their impact on the alcohol-related burden at ERs.

## Methods

### Sampling and setting of the study

A cross-sectional study of alcohol-related attendances at ERs was carried out in 11 ER of 11 hospitals of an urban area in Germany during a 6-months period (01 Sept 2000 – 28 Feb 2001). The study region was the city of Dortmund, with 587,000 inhabitants [17]. Through the initiative of the Dortmund City Council of Public Health all existing ERs in 11 hospitals serving the population of the city were included. The public health relevant purpose of this study was to obtain a complete assessment of the alcohol-related burden in a large city that was realized through a concerted initiative of all existing hospitals driven and led by the City Council of Public Health.

3,144 alcohol-related records were registered during the 6 months study period. Of these attendances about 10% (N = 314) across the study hospitals were *direct *admissions to inpatient units bypassing ER either via clinics or for pre-booked investigations. Our study focussed on the other 90% of alcohol-related attendances (N = 2,830) presented to the ERs first and that were contributed by 2,085 single patients for whom clinical charts were filled. In 310 (14.9%) of these patients the clinical charts were unusable, non-interpretable or completely missing and in further 27 patients (1.3%) the linkage of clinical and administrative information was impossible, leaving 1,748 (83.8%) single patients for this analysis.

### Inclusion criteria

Independently from the type of alcohol diagnosis all patients with either clinical symptoms of alcohol consumption or withdrawal syndrome presenting at ERs of the study region were eligible for this study, even considering patients with alcohol use several hours before presenting to ERs or patients who left ERs after a short-stay.

### Data sources

Two sources of data (clinical and administrative data) were combined for each individual patient. First, at ER the attending physician filled a chart on clinical routine data (clinical characteristics and diagnoses, degree of alcohol consumption, trauma, mode of referral and discharge, whether patient was accompanied by relatives or friends, and need for inpatient treatment) for each patient meeting the inclusion criteria. Second, administrative data (length of stay in the hospital, number of attendances, time and day of attendances and health care insurance) on each patient included into the study were obtained.

The clinical data were linked to the administrative data by the administrative departments and only anonymous data were further processed by the study group. Clinical data were entered on a sheet containing the patient's name before given to the administrative department who generated and entered an anonymous code on the sheet. The individual identifier (name and year of birth) on the sheet was then removed by the administrative department before the sheet was further processed by the study group. The administrative department destroyed the identifier and the list containing code and patient's name and year of birth. The study group received a blinded clinical data sheet which was entered into a data base. The administrative department provided for the study group an electronic data file containing administrative data on the hospital stay. The anonymous code on the clinical data sheet and the administrative data was used to merge both data sets.

While written informed consent was not able to be obtained, the information used was routine clinical and administrative data, which was provided for our use by the hospitals after patients had signed release of information forms.

### Clinical measures

The level of alcohol consumption was assessed objectively with the same model of alcometer (breathalyser Model: "Draeger, Alco-Test, 7410 ^plus^") across all hospitals and subjectively by the study physicians scoring each patient for the degree of alcohol consumption based on operational clinical criteria comprising 4 grades which were classified according to common clinical features related to alcohol consumption:

(1) withdrawal symptoms: physical / vegetative/psychological signs;

(2) low grade: inebriated, beginning of inhibitions, logorrhoea, euphoria, reduced concentration and attention, reduced self-criticism, alterations of balance;

(3) middle grade: increased intensity of symptoms of (2) plus beginning of abasia, blabber, reduced control over action;

(4) severe intoxication: successive increase of symptoms of (3) plus euphoria or depression, early signs of somnolence, loss of control;

Clinical alcohol diagnoses of acute alcohol intoxication, alcohol misuse, alcohol dependence and alcohol withdrawal were classified according to the criteria of the International Classification of Diseases, version 10 (ICD-10).

To make the study feasible in 11 hospitals over a period of 6 months no further information on e.g. drinking habits or number of drinks were obtained. Training of medical staff ensured the consistent use of operational clinical criteria of alcohol consumption or withdrawal and the use of breathalyser across all study hospitals. The clinical judgement of the degree of alcohol consumption was in good agreement with the alcohol breath test (Spearman-rho = 0.78). The inter-rater reliability (measured with Spearman's correlation coefficient, rho) showed values from rho = 0.87 – 0.53 across 11 hospitals and the intra-rater reliability had a similar range from rho = 0.87 – 0.62.

### Emergency rooms

Each study hospital provides typically for the German health care system a 24 h service of emergency care to patients. Patients attending an ER present with the need of acute medical care. Doctors at ERs provide acute treatment and make the decision on what type of further treatment is needed and whether patients are either treated on an outpatient level or admitted to a ward. In contrast, medical clinics serve patients who have pre-booked regular appointments and who are not an emergency case.

Typically, the study hospitals within the single city where the study took place do not provide the whole range of medical specialties. Thus, the ERs in this study are part of the hospitals and serving the medical specialties of internal medicine, surgery, addiction, and general psychiatry of the respective hospital. However, the largest hospital of the city, which serves as a tertiary hospital, provides an emergency service to all medical specialties of that hospital.

### Statistical analysis

The incidence of alcohol-related attendances at ERs and the number of inpatient-days related to these attendances at ERs was calculated for the six months study period and extrapolated for a 12 months period. For the calculation of the incidence all single and multiple attendances at ER during the study period were included.

All other analyses cover data from the study period of 6 months only. In the further analyses we allowed for only one attendance at ERs per patient (the first attendance for patients with multiple attendances). To make feasible an age comparison of the study sample with the general population of the City of Dortmund, age was classified into age groups according to the official statistics for the City of Dortmund.

Pearson's Chi^2 ^test was used to compare proportions of categorical data (e.g., gender differences, medical specialties, etc.). Separate binomial logistic regression models were applied to measure the relationship between multiple attendances, short-stay cases, respectively, and the degree of alcohol consumption and several other clinical characteristics (tables 2, 3). In separate multinomial logistic regression models we calculated Odds ratios for the association between four clinical degrees of alcoholic consumption (patients with alcohol withdrawal syndrome acted as a reference group) and demographic and clinical characteristics of patients presenting to ER for the first time during the study period (table 4). The continuous flow of the average number of patients through a 24 hours cycle of the day and a cycle of a typical week are presented in figures 2 and 3. All analyses were performed using SPSS-software, version 11 [18].

**Table 2:** Association of level of alcohol consumption with single and multipleattendances among 1,748 patients on first attendance at ERs

	**Single attendance (N = 1,451 patients)**	**Multiple attendances (N = 297 patients)**	**OR for multiple attendances**
**Factors**	**%**	**%**	**OR ^a ^(95% CI)**

**Gender ^b^**			
Male (N = 1,313)	57.7	42.3	1.95 (1.57–2.44)
Female (N = 404)	72.6	27.4	1 (reference)
**Age ^c^**			
≤ 20 years (N = 58)	98.3	1.7	1 (reference)
21 – 44 years (N = 907)	80.6	19.4	13.3 (1.8–96.5)
45 – 59 years (N = 568)	82.2	17.8	12.1 (1.7–88.1)
60 – 64 years (N = 103)	85.6	14.4	9.5 (1.2–73.6)
≥ 65 (N = 77)	96.1	3.9	2.3 (0.2–22.9)
**Clinical degree of alcohol consumption**			
High (N = 531)	55.0	45.0	2.62 (1.84–3.72)
Middle (N = 796)	62.6	37.4	1.94 (1.35–2.77)
Low (N = 231)	65.8	34.2	1.77 (1.19–2.65)
Symptoms of withdrawal (N = 190)	77.3	22.7	1 (reference)
**Results of alcohol breath test in millilitres**			
> 400 (N = 32)	52.4	47.6	2.30 (1.17–4.50)
201 – 400 (N = 474)	51.1	48.9	2.38 (1.79–3.18)
101 – 200 (N = 968)	59.9	40.1	1.70 (1.21–2.38)
≤ 100 (N = 274)	72.4	27.6	1 (reference)

^a ^separate logistic regression models calculated for each factor showing Odds ratios for multiple attendances, adjusted for age and sex; OR denotes Odds ratio; CI denotes confidence interval; ^b ^31 missing values; ^c ^33 missing values;

**Table 3:** Association of clinical characteristics and duration of stay < 24 h among 1,748 patients at first alcohol-related attendance at ERs

	**Duration of stay ≥ 24 h (N = 1,148 patients)**	**Duration of stay <24 h (N = 600 patients)**	**OR for duration of stay < 24 h**
**Clinical factors**	**(%)**	**(%)**	**OR ^a ^(95% CI)**
**Repeat attendance**			
Yes (multiple), (N = 297)	56.3	43.7	1.24 (1.04–1.49)
No (single) (N = 1,451)	63.3	36.7	1 (reference)
**Clinical degree of alcohol consumption**			
High (N = 531)	44.3	55.7	9.39 (5.63–15.64)
Middle (N = 796)	58.4	41.6	5.47 (3.28–9.14)
Low (N = 231)	71.8	28.2	3.04 (1.73–5.35)
Withdrawal (N = 190)	88.8	11.2	1 (reference)
**Discharge mode ^b^**			
Self-discharge (N = 207)	58.8	41.2	1.33 (0.97–1.81)
Medical discharge (N = 1,173)	66.0	34.0	1 (reference)
**Breath test (millilitres)**			
>400 (N = 32)	48.4	51.6	6.7 (2.9–15.0)
201–400 (N = 474)	60.7	39.3	4.1 (2.7–6.3)
101–200 (N = 968)	55.6	44.4	5.2 (3.5–7.8)
≤ 100 (N = 274)	86.9	13.1	1 (reference)
**Alcohol diagnosis at ER attendance ^c^**			
Misuse (N = 497)	33.9	66.1	11.56 (7.67–17.43)
Dependence (N = 924)	68.3	31.7	2.81 (1.91–4.13)
Withdrawal (N = 300)	86.2	13.8	1 (reference)
**Diagnosis or suspicion of trauma ^d^**			
Yes (N = 263)	37.6	62.4	3.4 (2.51–4.60)
No (N = 1,383)	65.5	34.5	1 (reference)

^a ^separate logistic regression models calculated for each clinical factor showing Odds ratios for the duration of stay below 24 h, adjusted for age and sex; ^b ^the category "referral to other hospital" was not considered in this table; see table 1 for information on missing values about mode of discharge; ^c ^missing information on diagnosis among 27 cases; ^d ^missing information on trauma among 102 cases; OR denotes Odds ratio; CI denotes confidence interval;

**Table 4:** Demographic and clinical factors associated with various degrees of alcohol consumption for 1,748 patients presenting at ERs for the first time during 6 month study period (OR ^a^; 95% CI)

**Categories**	**Gender**^b^	**Duration of stay**	**ER attendance^c^**	**ER attendance^d^**	**Trauma^f^**	**No of attendances**	**Discharge mode^g^**
**Clinical degree/signs of alcohol consumption**	Male (N = 1,313) vs. Female (N = 404)	<24 h (N = 600) vs. = 24 h (N = 1,148)	Weekends (N = 1,234) vs. Weekdays (N = 479)	Outside (N = 867) vs. Regular (N = 820) working time^e^	Yes (N = 263) vs. No (N = 1,383)	Multiple (N = 297) vs. Single (N = 1,451)	Self-discharge (N = 207) vs. Medical discharge (N = 1,171)

High (N = 531)	2.21 (1.51–3.23)	9.42 (5.65–15.70)	1.73 (1.16–2.59)	8.56 (5.72–12.81)	3.17 (1.72–5.83)	2.62 (1.84–3.73)	1.93 (1.08–3.42)
Middle (N = 796)	1.61 (1.11–2.32)	4.93 (3.29–9.18)	1.39 (0.93–2.09)	6.38 (4.29–9.51)	3.52 (1.92–6.46)	1.94 (1.36–2.78)	1.59 (0.89–2.85)
Low (N = 231)	1.28 (0.84–1.95)	3.04 (1.73–5.35)	1.49 (0.95–2.36)	2.09 (1.33–3.27)	2.39 (1.22–4.71)	1.78 (1.19–2.66)	1.16 (0.57–2.35)
Withdrawal (N = 190)	1.0 (reference)	1.0 (reference)	1.0 (reference)	1.0 (reference)	1.0 (reference)	1.0 (reference)	1.0 (reference)

^a ^separate multinomial regression models calculated for each category showing Odds ratios for the degrees of alcohol consumption, adjusted for age and sex;

^b ^31 missings; ^c ^34 missings^; *d *^52 missings; ^e ^regular time: 8 a.m. – 4 p.m.; ^f ^102 missings; ^g ^the category "referral to other hospital" was not considered in this analysis; see table 1 for information on missing values about mode of discharge; OR denotes Odds ratio; CI denotes confidence interval;

**Figure 1:**
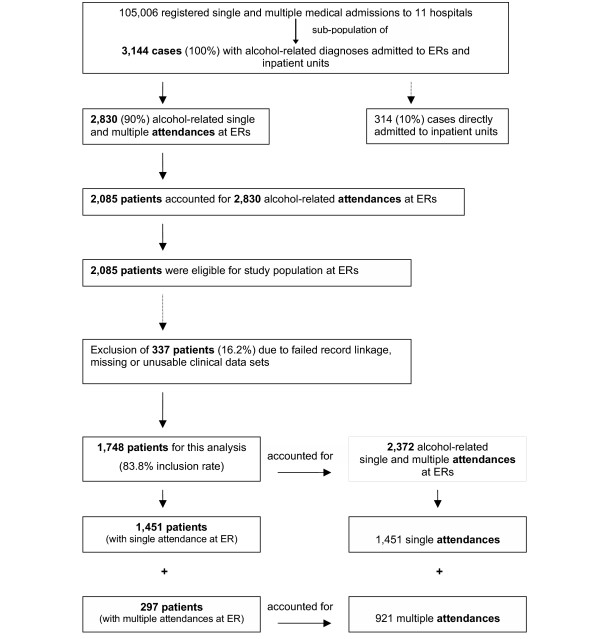
Sampling and study population.

**Figure 2:**
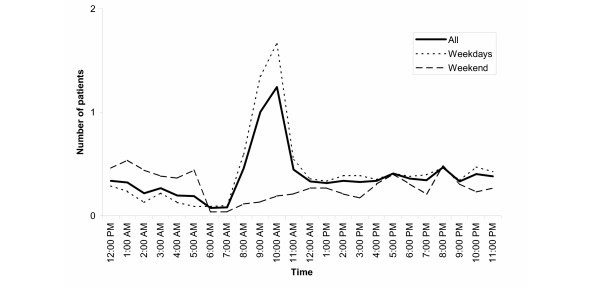
**Flow of average number of patients during course of the day by time during the weekdays and at weekends. **Note 1: based on 1,696 patients (52 missing) for whom the exact time of attendance was available

**Figure 3:**
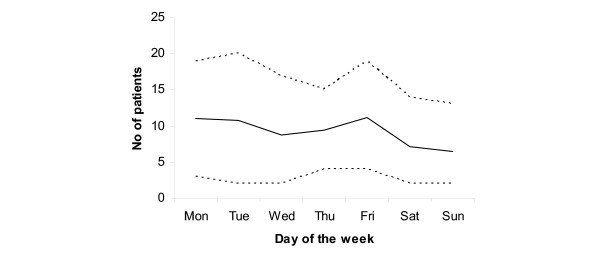
Average number of patients on different days of the week (Mon-Sun). Note 1: line – average number; scatter above line – 95^th ^percentile; scatter below line – 5^th ^percentile. Note 2: based on 1,714 patients (34 missing) for whom the exact date of attendance was available

## Results

### Study population

Overall, during the 6 months study period there were 105,006 medical admissions (annual incidence of 358 per 1,000 inhabitants) to the 11 study hospitals. These admissions contained 3,144 alcohol-related attendances (3%) to ERs *plus *direct admissions to inpatient units due to any type of primary or secondary alcohol diagnosis. These 3,144 alcohol-related attendances (including multiple attendances) and admissions yielded an extrapolated annual incidence of 10.7 cases per 1,000 inhabitants.

A total of 1,748 patients (75.1% male) attended ERs during the study period after the exclusion of 337 patients with either missing or non-interpretable (16.2% of the 2,085 eligible cases) charts (Figure 1). These patients contributed 2,372 alcohol-related attendances (male to female ratio: 3.85:1) to ERs. In total, 1,959 of all attendances (82.6%) led to inpatient admission resulting in 12,629 days of inpatient treatment (6.5 days on average per admission to inpatient units). The average duration of inpatient stay was 10 days (95% CI, 9.5–10.5; min. 1; max. 108; SD 0.26; median 9).

### Demographic and clinical characteristics of the study population

About one quarter of the patients was female (23.1%) and the median age of all patients was 43 years (min: 14y; max: 85y; mean: 43.3y; SD: 0.29). Mean age of men was significantly lower than of females (42 years, SD 11.9 vs. 44 years, SD 12.8; Student-t-test p < 0.01). In comparison to the general population of the City of Dortmund we observed some systematic differences in age and gender for the study population. While about 3.3% of the patients were = 20 years of age, this age group accounted for 20.3% of the general population. Whereas 19% of the general population was 65 years of age or older only 4.4% patients were of that age in our study. As shown in Table 1 males were predominant in this study, while the male:female ratio in the general population was 0.9:1.

**Table 1:** Demographics and characteristics of patients (N = 1,748) on first alcohol-related attendance at ERs in this study

	**Study population N = 1,748**
**Age groups^a^**	%
≤ 20 years	3.3
21 – 44 years	51.9
45 – 59 years	32.5
60 – 64 years	5.9
≥ 65 years	4.4
Missing	1.9
**Gender**	
Male	75.1
Female	23.1
Missing	1.8

**Medical specialties associated with ER**	
Internal medicine	46.7
Addiction	25.5
Mixed medical disciplines	15.2
Surgery	6.5
General Psychiatry	5.7
Other	0.4
Mode of discharge	
Regular	67.1
Own request, against medical advice	11.8
Referral to other hospital	3.0
Missing ^b^	18.1 ^b^

^a ^Age of study sample is grouped according to the official statistics of the City Council Dortmund, for the purpose of group comparison in this analysis.

^b ^65% of these patients stayed less than 24 h in hospital and 58% of these were not referred to inpatient units.

At attendance 53.7% of the patients presented with dependent alcohol use, 28.9% with non-dependent alcohol use and 17.5% with an alcohol withdrawal syndrome. About 94.5% of the patients with initial withdrawal syndrome were classified as dependent alcohol use at discharge. About 15% of the patients had a trauma or suspicion of a trauma at ER attendance.

### First-time ER attendances in several medical specialties

The majority of the first-time attendances took place at ERs of internal medicine (46.7%) and addiction (25.5%), less often at ER serving mixed disciplines based at a general hospital (15.2%), and marginally at ERs providing surgery (6.5%), general psychiatry (5.7%), and others (0.4%).

Next, we compared repeat cases to single cases and found that the proportion of repeat cases was significantly highest at the ER of the clinic of addiction (21.6%), secondly at the ER serving mixed disciplines (19.3%), followed by the specialty of general psychiatry (12.7), the ERs serving internal medicine (12.6%) and ERs for surgery (5.6%), (Chi^2^-test: p < 0.0001).

We also compared the proportion of cases staying ** = **24 hours to cases staying <24 hours, and observed that the proportion of short-stays was highest at the ER serving mixed disciplines (95.2%), then at ERs serving surgery (42.0%), internal medicine (16.9%), general psychiatry (14.8%) and clinic of addiction (1.1%), (Chi^2^-test: p < 0.0001).

The proportion of patients with high degree of alcohol consumption was highest at ERs serving mixed disciplines (72.9%), then at ERs serving surgery (46.9%), followed by general psychiatry (37.7%), and ERs of internal medicine (31.8%), and was lowest at the clinic of addiction (27.7%), (Chi^2^-test: p < 0.0001).

### Gender specific clinical characteristics

Females were significantly (p < 0.05) more likely to present with medical referral papers from a physician, to have a lower degree of alcohol consumption, to stay longer than 24 hours in hospital, to present once only at ER in 6 months and to be classified more often with the diagnose of alcohol withdrawal syndrome instead of non-dependent or dependent alcohol use compared to their male counterparts.

Furthermore, women were significantly (Chi^2^-test: p < 0.05) more often accompanied by friends or relatives and were less likely to use ambulance services for transportation, but they did not differ significantly from male patients in the proportion of inpatient treatment following attendance at ER, mode of discharge and having trauma or suspicion of a trauma.

### Attendances at ERs by time and weekday

Variations of attendances at ER by time of the day and weekday may reveal peaks and lows of the alcohol-related clinical burden at ER indicating special need for personnel and other resources. Given the exact time of attendance, the flow of the average number of patients through a 24 hour cycle is presented in figure 2. Figure 3 shows the average number of patients per weekday through a 1 week period. On average, there were 9.7 attendances per day with some systematic variation between different weekdays and between different hours of the day (Figures 2 and 3). During weekends there was no time preference for attendances but during weekdays most attendances occurred between 8 a.m. to 4 p.m. Almost 28% of all attendances occurred on weekends (Sat. + Sun.). A decrease in attendances during winter months was observed.

### Single vs. repeat attendance at ERs

In total, 1,451 (83%) patients were admitted once during the study period and 297 patients contributed the remaining 921 multiple attendances (min. 2; max. 63; mean 8; SD 0.5; median 3). Whereas most of the patients (76%) with repeated attendances had up to 5 attendances there were 10% with 27 or more attendances during the study period (3.2% had more than 45 attendances). Patients with multiple attendances (17% of all patients) were responsible for 39% of all attendances at ER indicating a huge clinical burden caused by this group.

Thus, we further looked at factors associated with multiple attendances. Patients aged between 31–50 years, patients non-fixed abode, and males were significantly more likely to present multiple times than younger or older patients (Chi^2^-test: p < 0.05).

Table 2 shows factors associated with multiple attendances at ERs. Separate logistic regression models revealed statistically significant increased odds for the association of multiple attendances with male gender, higher clinical degrees of alcohol consumption and higher alcohol concentrations assessed by the breath test. The latter two variables indicated dose-response effects between alcohol consumption and multiple attendances at ERs. High degrees of alcohol consumption at first ER attendance predicted repeat attendance in due course.

### Factors associated with duration of stay at hospital

Duration of stay (< 24 hours versus = 24 hours) at ERs and hospital, respectively, gives an indication whether patients with alcohol-related problems presenting at ERs finally receive inpatient treatment and whether or how clinical and demographic characteristics differ among these groups.

About one third of the patients left hospital within 24 hours (40.8% males vs. 29.3% were females, Chi^2^-test: p < 0.0001), another third stayed between 1–7 days (19.6% males vs. 26.8% females, Chi^2^-test: p < 0.0001) and the remaining third of patients stayed for more than 7 days (39.6% males vs. 43.9% females, Chi^2^-test: p < 0.0001). The average length of stay was 10 days (95% CI, 9.5–10.5; min: <1 day; max: 108 days; SD 0.26). The length of stay varied by age: while we found 15 days on average for patients below 20 years of age, we observed 7.4 days on average for the 21–30 years old. The remaining age groups had a similar average duration of stay between 9–10 days.

Males, patients below the age of 30 years and above the age of 50 years, patients with no fixed abode and patients supported by social services were more likely to leave the hospital within 24 hours (Chi^2^-test: p < 0.05). Although most patients (79.7%) in this sample were admitted to inpatient units, about one third (31.1%) of them stayed less than 24 hours in hospital. While few of them self-discharged against medical advice (13.8%), the majority of (86.2%) left hospital within 24 h after regular medical discharge or transfer.

Table 3 presents clinical characteristics associated with short duration of stay (<24 hours) for patients with first alcohol-related attendance at ERs during study period. Statistically significant associations between short stay (<24 hours) at ERs were found for male gender, multiple attendances, patients with trauma or suspicion of trauma, alcohol dependence or alcohol misuse. Dose-response effects were seen for higher degrees of alcohol consumption assessed by clinical judgement and breath-test on short duration of stay. Self-discharge was not significantly associated with short duration of stay (Table 3).

### Factors associated with clinical degree of alcohol consumption

Higher degrees of alcohol consumption mostly indicate the need of intensified medical care and often limit the motivational or psychological access to patients. Therefore we were interested in factors associated with the clinical degree of alcohol consumption of patients at ER.

Clinical categories were analyzed for their association with different degrees of alcohol consumption (according to the 4 clinical degrees of alcohol consumption) with separate (for each category) multinomial logistic regression models. We found statistically significant associations for higher degrees of alcohol consumption with male gender, trauma (or suspicion of trauma), duration of stay <24 hours, multiple attendances, discharge against medical advice, attendances on weekends and attendances outside regular working times (Table 4).

Significant linear dose-response relationships were seen for the effects of short duration of stay, multiple attendances, attendances outside regular working times, discharge against medical advice and male gender on increasing clinical degree of alcohol consumption on (Table 4). Age alone was without significant association with any of the clinical degrees of alcoholic consumption, neither as continuous nor as categorical variable (data not shown).

## Discussion

In this study the demographic characteristics, clinical burden and profile of alcohol-related attendances at ERs was analyzed for 1,748 patients in 11 hospitals of an urban area. With this study design we considered a broad definition of alcohol-related attendances independently from alcohol diagnosis, different hospital settings and a wide range of medical specialties over a period of 6 months which is an advantage over previous studies in this field which concentrated on single settings or specialties,[7,8]. Since all hospitals of the urban region were involved in this study, the obtained estimates can be related to the population of the area. The prospective design of this study allowed for obtaining information on characteristics of single compared with repeat attendances.

Instead of tests such as CAGE, the Michigan Alcoholism Screening test (MAST) and the Munich Alcoholism Test (MALT) [19-21] we applied an objective measure of alcohol consumption using breath test and a standardized clinical measure. For the purpose of this study to estimate the alcohol-related clinical burden at ERs independently of specific alcohol diagnoses we found the applied criteria to be valid and feasible measures.

The proportion of alcohol-related attendances at ERs in relation to all medical attendances / inpatient admissions was 3% in this study. This figure is lower than in other studies such as in Liverpool, UK: Pirmohamed et al. reported a proportion of all alcohol-related hospital admissions of 6.2% [14] that is line with figures from the National Statistics for England and Wales (1 alcohol case in 16 hospital admissions) [22]. The proportion of 3% alcohol-related admissions in our study was twice as high as the corresponding figure of the National German Hospital Statistics (1 alcohol case in 64 hospital admissions), [23] indicating an over-representation of alcohol-related attendances and admissions to hospitals of the study region.

The high proportion of male gender in this sample reflects the well known greater likelihood of alcohol-related diagnoses for men than for women in the general population [24,25] and in clinical samples [26] as well. Female gender was associated with social behaviour better adapted to regular working times at ERs, regular medical procedures and with more social support by friends.

Patients of the age groups 21–44 years and 45–59 years with alcohol problems at ERs were over-represented in this study compared to the age distribution in the general population of Dortmund implicating intensive and dangerous alcohol consumption in these age groups. However, age was neither significantly associated with higher degrees of alcohol consumption nor with non-dependent alcohol use in this sample. This is controversial in light of findings reported in the German annual report on alcohol and drug use in the general population. They found an increase of hazardous alcohol consumption (women >20 g alcohol daily; men >30 g alcohol daily) in younger people aged 18–24 between the years 1995–2001 and a slight decrease in older men and women in the general population [27]. The cross-sectional findings in our study did not mirror these age-related national trends for the year 2001 (neither for men nor for women). Our finding is also contrary to studies in Ireland reporting highest rates of non-dependent alcohol use among the 20–29 years-old [15] and 18–29 years-old [28], respectively. O'Farrell et al. reported in an Irish study that 41% of the acute alcohol intoxication admissions to inpatient units of general hospitals were in young people under 30 years of age [15]. In our study this age group contributed only 13% attendances. Even when we restricted our analysis to the subgroup of patients with inpatient stay, the proportion changed only slightly.

However, the proportion of patients with alcohol use and their related pattern of alcohol misuse may differ depending on samples from the general population, in primary care and emergency rooms as reported by Cherpitel et al. [29].

The proportion of patients with short stay was 34.3% in this study, about 10% higher than the proportion of short stays in a similar study by O'Farrell et al. in Ireland [15]. This difference is probably due to the fact that O'Farrell et al. captured only inpatient hospital admissions (>24 hours attendance) in contrast to our study that also captured short stays less than 24 hour attendance. Another difference between these studies was related to the average duration of hospital stay (10 days in this study) that was more than 3 times higher than in O'Farrell's study (2.3 days on average) indicating a huge effect on the hospital resources [15]. These differences mirror the generally longer duration of stay of patients in inpatient units in Germany (nationwide 9.3 inpatient days on average in general hospitals) compared to the UK (nationwide 5.5 inpatient days on average in general hospitals in NHS beds) for the year 2001 [14,22,30].

In our study higher degrees of alcohol consumption were associated with attendances at ERs on weekends which is similar to findings reported in a study on emergency inpatient hospital admissions in Ireland [15]. The same effect was seen for patients with attendances at ERs outside regular working times. Both effects may reflect the harmful drinking pattern that occurs over the weekend period and during evening and night. Since harmful drinking patterns on weekends and at night time are found to be associated with alcohol-related traffic accidents in Germany [16], these patients presenting at ER are at higher risk to be involved than non-alcoholics.

Our results show that short-stay cases and cases with high degrees of alcohol consumption represent high risk groups for potential alcohol-related medical complications (e.g., trauma). Although no figures are available showing the proportion of patients involved in alcohol-related road accidents after discharge from ERs, especially short-stay cases and cases with higher degrees of alcohol consumption may represent a group with higher risk to themselves and in road traffic. A study by Cryer et al. in the general population supported the generally held view that heavy alcohol consumers are disproportionate users of acute medical services, but they are relative under-users of preventative medical care services [31]. The short-stay cases in our study would fit into this pattern of service use as they tend not to utilize the inpatient treatment facilities.

The findings in this study on the association of trauma with higher degrees of alcohol consumption confirm previous findings on this issue. Cherpitel et al. reported in a meta-analysis a moderate, but consistent association between higher blood alcohol concentration and injury in emergency room settings [32,33].

### Limitations of the study

We reached the goal to include all ERs of the study region. However, due to possible moving of the patients outside and within the catchment area the study population was an open cohort. A proportion of 18% of the patients were not registered as living in the catchment area, and we did not obtain information if they moved into this area for the short-term. It remained unclear whether these patients are balanced by patients registered as living in the study region but presenting to ERs in other regions. Moreover, the proportion of people officially registered in the study area is unknown and also unknown is the proportion in the sub-sample of inhabitants with alcohol-related problems. Furthermore, patients also could have presented in two different study hospitals during the study period. Given the size of the city and the wide spread locations of the hospitals (one central hospital, several peripheral district hospitals, and hospitals in the outskirts of the town), we assumed multiple presenting of the same patient in different hospitals was not very likely in this study, because the study hospitals are not in easy reach of each other. All these effects could lead to either over- or underestimation of incidence in our study.

Because of the relatively short time (from September to February) covered by the study, we do not know the number of patients in the spring and summer months (March-August). The observed decrease of attendances at ERs during winter months could be explained by a diminished recruitment in the course of the study, but can also result from climatic conditions. On the other hand, based on official statistics on drinking patterns in Germany there is no indication of an increasing or declining trend of incidence of alcohol-related attendances at ER during the Christmas holiday period [27]. Given the observed trend in this study is true, our estimates of incidence of attendances at ERs and days of hospitalisation are rather too low.

We obtained no information on patients not included in the study. If particular characteristics were associated with the non-inclusion status, our estimates of associations could be distorted, however, the relatively high inclusion rate of 83.8% makes a strong influence of missing cases improbable.

## Conclusion

Although the figure obtained in this survey is likely to be an underestimate of the true alcohol-related burden in hospitals, the results demonstrate a substantial need for tailored interventions at ERs. The presented trends on patterns of alcohol use in the general population support this view. In particular, short-stay cases, repeat attendances and cases with higher degrees of alcohol consumption largely determine the clinical burden at ERs serving several medical disciplines. Since suitable brief anti-alcoholic interventions have been proven to initiate change of drinking patterns [1,34,35] and have demonstrated their effectiveness in patients with alcohol problems in primary care settings [36-39] and in emergency departments as well [40], the design, implementation and evaluation of anti-alcoholic interventions applied to ERs would need to take into account the factors determining the alcohol-related clinical burden presented in this study.

## Competing interests

The author(s) declare that they have no competing interests.

## Authors' contributions

BB conceived the study, guided the statistical analysis and drafted the manuscript. RM performed the statistical analysis and contributed to drafting of the manuscript. GR conceived the study, designed and developed the questionnaire and co-ordinated the data collection in the addiction hospital. AD conceived the study and co-ordinated the data collection in the general hospitals. SF developed and tested the questionnaire, applied the questionnaire across the hospitals and carried out data collection and data management. HK planned the study and organized clinical and administrative data collection. US supervised data collection and data management throughout the study period. All authors revised and approved the final manuscript.

## Pre-publication history

The pre-publication history for this paper can be accessed here:


